# The impact of cyclin D1 overexpression on the prognosis of bladder cancer: a meta-analysis

**DOI:** 10.1186/1477-7819-12-55

**Published:** 2014-03-06

**Authors:** Baoming Ren, Wenjun Li, Yanping Yang, Songdi Wu

**Affiliations:** 1 Department of Urinary Surgery, First Hospital of Xi’an, Xi’an, Shaanxi 710002, China; 2 Department of Medical Technology, Shaanxi Energy Institute, Xi’an, Shaanxi 712000, China; 3 Department of Neurology, First Hospital of Xi’an, Xi’an, Shaanxi 710002, China

**Keywords:** Bladder cancer, Cyclin D1, Meta-analysis, Prognosis

## Abstract

**Background:**

To evaluate the relationship between cyclin D1 overexpression and bladder cancer prognosis.

**Methods:**

A systematic literature search up to July 27, 2013 was carried out in PubMed and Wanfang databases, and the references of retrieved articles were screened. The hazard ratios (HRs) and their 95% CIs were used to combine the data. Heterogeneity and publication bias were also evaluated.

**Results:**

A total of 15 studies containing 2,591 cases were included. We found that increased cyclin D1 levels were significantly correlated with progression-free survival with a pooled HR estimate of 0.54 (95% CI: 0.32–0.92). There was a significant degree of heterogeneity (I^2^ = 93.8%, *P* <0.001). Moreover, subgroup analysis indicated that elevated cyclin D1 levels were significantly associated with overall survival in muscle-invasive bladder patients (HR: 0.95, 95% CI: 0.91–0.99), without a significant heterogeneity in the data (I^2^ = 0.0%, *P* = 0.456).

**Conclusions:**

Increased cyclin D1 expression level detected by immunohistochemistry is associated with good progression-free survival for bladder cancer. Because of the limited number of studies, further well-designed prospective studies are warranted to confirm the findings from our study.

## Background

Urothelial bladder cancer ranks ninth in worldwide cancer incidence; it is the seventh most common malignancy in men and seventeenth in women [1]. Despite advances in treatment, the prognosis of bladder cancer, especially muscle-invasive tumors, remains poor. An estimated 386,300 new cases and 150,200 deaths from bladder cancer occurred in 2008 worldwide [2].

Recently, increasing attention is being paid to the prognostic biomarkers in bladder cancer patients. Although genes associated with epithelial-mesenchymal transition (e.g., E-cadherin), apoptosis (e.g., p53), and angiogenesis (e.g., VEGF) have been investigated by several studies, the identification of a well-established marker possessing the predictive value for survival of bladder cancer patients remains a topic that needs to be explored [3-5].

Cyclin D1 (CCND1) is located on chromosome 11q13. As a key regulator of the G1 progression step within the cell cycle, it is a major positive regulator of the G1 restriction point [6]. Cyclin D1 expression is altered in various cancers, suggesting that its deregulation contributes to tumorigenesis. For bladder cancer, cyclin D1 also has been reported to play an important role in origin, development, and dissemination of the disease [7]. However, in previous studies, the association between tissue expression of cyclin D1 and carcinogenesis and/or clinical outcome remains inconclusive. Cyclin D1 protein expression has been reported to be correlated with both poor and good prognosis, partially since a single study might be too underpowered to detect a possible small effect of cyclin D1 expression on bladder cancer prognosis, especially when the sample size is relatively small. In this study, we conducted a systematic review and meta-analysis to estimate the effect of cyclin D1 altered expression on the survival of bladder cancer patients.

## Methods

### Publication search

We carried out a search in PubMed and Wanfang databases, covering all the papers published from their inception to July 27, 2013, using the following search algorithm: (cyclin D1 or CCND1) and (bladder cancer or bladder tumor or bladder neoplasm or urothelial cancer or urinary tract cancer) and prognos*. We evaluated potentially relevant publications by examining their titles and abstracts and all the studies matching the eligible criteria were retrieved. We also checked the references from retrieved articles and reviews to identify any additional relevant studies. This study was planned, conducted, and reported in adherence to the standards of quality for reporting meta-analyses [8].

### Inclusion criteria

Studies included in this meta-analysis had to meet all the following criteria: i) to evaluate the correlation between cyclin D1 expression and prognosis of bladder cancer patients; ii) to assess cyclin D1 expression in the primary tumor tissues using immunohistochemistry (IHC); and iii) to provide sufficient information allowing for estimation of hazard ratios (HRs) and their 95% confidence intervals (CIs). If multiple publications from the same study population were available, the most recent and detailed study was eligible for inclusion in the meta-analysis.

### Data extraction

Information was carefully extracted independently by two authors according to the inclusion criteria noted above. For each study, the following characteristics were collected: the first author’s name, year of publication, the country in which the study was carried out, sample size, age of patients, follow-up years, disease stage, cut-off value, increased cyclin D1 expression, and survival data.

### Quality assessment

The quality of each study was assessed by the same two investigators using the Newcastle-Ottawa Quality Assessment Scale for cohort studies with our reasonable modifications (see “Newcastle-Ottawa quality assessment scale” section). This scale is an eight-item instrument that allows for assessment of patient population and selection, study comparability, follow-up, and outcome of interest. Interpretation of the scale is performed by awarding points, or ‘stars’, for high-quality elements. Stars are then added up and used to compare study quality in a quantitative manner. The scores range from 0 to 9. We assigned scores of <7 and ≥7 for low and high quality of studies, respectively.

### Newcastle-Ottawa quality assessment scale

#### Selection

(1) Representativeness of the exposed cohort

(a) Truly representative of the average bladder cancer (BCa) patients in the community*

(b) Somewhat representative of the average BCa 2 patients in the community*

(c) Selected group of users (e.g., nurses, volunteers)

(d) No description of the derivation of the cohort

(2) Selection of the non-exposed cohort

(a) Drawn from the same community as the exposed cohort*

(b) Drawn from a different source

(c) No description of the derivation of the non-exposed cohort

(3) Ascertainment of exposure (*Proof of BCa and Cyclin-D1 measurement*)

(a) Secure record (e.g., surgical records)*

(b) Structured interview*

(c) Written self-report

(d) No description

(4) Demonstration that outcome of interest was not present at start of study

(a) Yes*

(b) No

#### Comparability

(1) Comparability of cohorts on the basis of the design or analysis

(a) Study controls for smoking*

(b) Study controls for any additional factor (Age, gender, grade, etc.)*

#### Outcome

(1) Assessment of outcome

(a) Independent blind assessment*

(b) Record linkage*

(c) Self report

(d) No description

(2) Was follow-up long enough for outcomes to occur? (Death or recurrence or progression)

(a) Yes (3 years)*

(b) No

(3) Adequacy of follow-up of cohorts

(a) Complete follow-up – all subjects accounted for*

(b) Subjects lost to follow-up unlikely to introduce bias – small number lost – (25%) follow-up, or description provided of those lost*

(c) Follow-up rate (≤75%) and no description of those lost

(d) No statement

A study can be awarded a maximum of one star (*) for each numbered item within the Selection and Outcome categories. A maximum of two stars can be given for Comparability. Underlined and quoted phrases are provided in the scale to allow for adjustment to particular studies. Italicized phrases indicate our interpretation of the question relevant to this study.

### Statistical methods

HRs and their 95% CIs were used to combine the data. When these statistical variables were described in text or tables, we obtained them directly from each trial publication. When not given explicitly in an article, they were calculated from available numerical data in the articles using methods reported by Parmar et al. [9]. In this meta-analysis, DerSimonian-Laird random effect analysis [10] was used, as a result of *a priori* assumptions about the likelihood for heterogeneity. By convention, an observed HR >1 implies worse survival for the group with positive/increased cyclin D1 expression. The impact of positive/increased cyclin D1 expression on survival was considered to be statistically significant if the 95% CI did not overlap with 1.

Homogeneity of ORs across studies was tested by a χ^2^-based Q statistic and the I^2^ score. Heterogeneity was considered significant if the *P* value is <0.10. The value of I^2^ is used to assess the degree of heterogeneity (I^2^ < 25% no heterogeneity; I^2^ = 25–50% moderate heterogeneity; I^2^ > 50% large or extreme heterogeneity).

### Evaluation of publication bias

Publication bias was assessed using Begg’s test (rank correlation method) [11] and Egger’s test (linear regression method) [12]. *P* <0.05 was considered to be representative of a significant statistical publication bias. All of the statistical analyses were performed with STATA 11.0 (StataCorp, College Station, TX, USA), using two-sided *P* values.

## Results

### Study selection and characteristics

**Figure 1:**
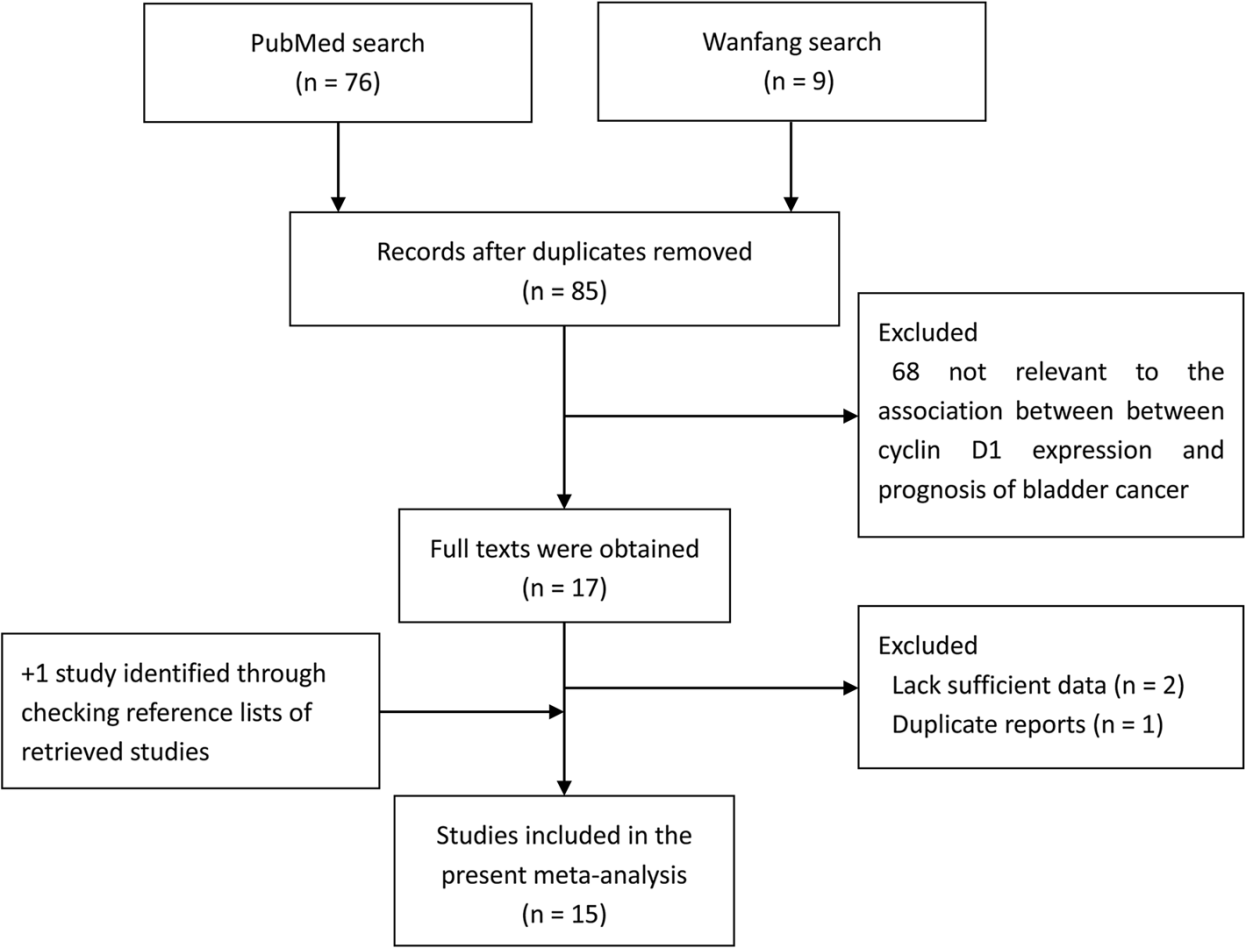
Process of study selection.

A total of 85 articles were identified from a search of the above databases using the search strategy as described above (Figure 1). After exclusion of the trials that were out of the scope of our systematic review, 17 studies assessing prognostic value for survival of cyclin D1 status in patients with bladder cancer were considered eligible for inclusion in the evaluation. Upon further review, 2 were excluded since it was not possible to allow for the calculation of HR estimate because of insufficient reported data, 1 was excluded because it had overlapped data with other studies, and 1 was identified through checking reference lists of retrieved studies. After selection, a total of 15 publications [13-27] were finally enrolled for analysis of the prognostic value of cyclin D1 expression in bladder cancer (Figure 1).

The clinical features of these 15 included studies (16 cohorts) eligible for the meta-analysis are summarized in Table 1; 2 studies evaluated patients from Japan, 2 from Korea, 2 from Italy, 2 from Spain, 1 from Finland, 1 from the UK, 1 from Switzerland, 1 from the USA, 1 from Greece, 1 from Sweden, and 1 from four countries (Denmark, Sweden, Spain, and Taiwan). The 15 studies comprised 2,591 patients, with sample sizes ranging from 74 to 576 patients (mean 162); 5 of these studies enrolled less than 100 patients and 3 studies included more than 200 patients. The follow-up period was at least 19 months, while 11 cohorts were followed-up for more than 3 years. HRs were recorded for each study using available data or the methods described above. Overall survival (OS) was reported in 10 studies, progression-free survival (PFS) was reported in 7 studies, recurrence-free survival (RFS) was reported in 4 studies, and disease-free survival (DFS) was reported in 2 studies. The points of study quality assessed by Newcastle-Ottawa quality assessment scale ranged from 5 to 7 (with a mean of 6.4).

**Table 1:** Main characteristics of all studies included in the meta-analysis

**Study**	**Year**	**Country**	**Mean/median age**	**Sample size**	**Follow-up**	**Stage**	**Cut-off (%)**	**Positive (%)**	**Quality score**	**Survival analysis**	**Hazard ratios**
Shin et al. [13]	1997	Korea	62	75	35	All	5	50.7	5	OS	Estimated
Liukkonen et al. [14]	2000	Finland	65.8	187	58.8	Superficial	10	63.6	7	PFS	Estimated
Takagi et al. [15]	2000	Japan	69.3	102	41	All	NR	77	7	OS	Estimated
Tut et al. [16]	2001	UK	68	150	33	All	8	83	5	OS	Estimated
Sgambato et al. [17]	2002	Italy	68	96	50	Superficial	25	52.1	7	OS, RFS, DFS	Estimated
Lopez-Beltran et al. [18]	2004	Spain	61	159	74.8	Superficial	15	33.3	7	OS, DFS	Estimated
Mhawech et al. [19]	2004	Switzerland	70.3	101	19	Superficial	10	53.5	6	PFS	Reported in text
Galmozzi et al. [20]	2006	Italy	NR	82	21	Muscle-invasive	10	64.6	6	OS	Reported in text
Yurakh et al. [21]	2006	Spain	NR	84	36.4	All	10	NR	7	OS, PFS	Reported in text
Shariat et al. [22]	2007	USA	63.2	74	42.3	Superficial	30	68.9	7	OS, RFS, PFS	Estimated
Lee et al. [23]	2010	Korea	67	103	31.5	All	10	29	6	OS	Reported in text
Levidou et al. [24]	2010	Greece	69	157	44.95	Muscle-invasive	40	NR	5	OS	Reported in text
Behnsawy et al. [25]	2011	Japan	NR	161	47	Superficial	20	24.8	7	RFS	Reported in text
Olsson et al. [26]	2012	Sweden	73	201	60	Superficial	10	71	7	RFS, PFS	Reported in text
Fristrup et al. A [27]	2013	Denmark	68	283	103	Superficial	20	NR	7	PFS	Reported in text
Fristrup et al. B [27]	2013	Sweden, Spain, and Taiwan	71	576	80	Superficial	20	NR	7	PFS	Reported in text

NR, not reported; OS, overall survival; RFS, recurrence-free survival; PFS, Progression-free survival; DFS, Disease-free survival; “Fristrup et al. A” here means the data in this row from Fristrup et al. [27] training cohort, “Fristrup et al. B” means the data in this row from Fristrup et al. [27] validation cohort; Study quality is listed using the results of the Newcastle-Ottawa quality assessment scale.

### Cyclin D1 expression and OS in bladder cancer

**Figure 2:**
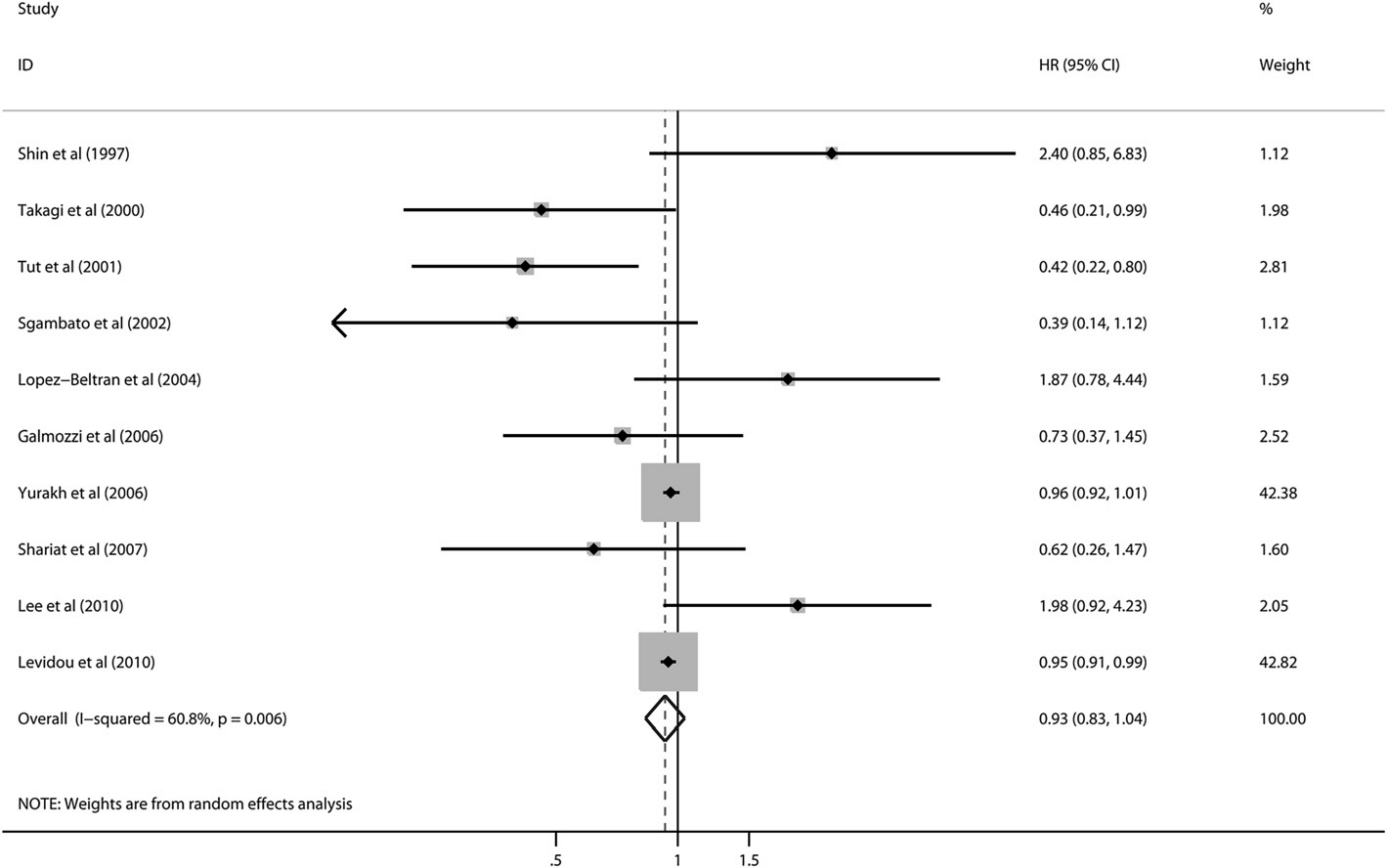
Hazard ratios (HRs) and 95% confidence intervals (CIs) in studies assessing the relationship between cyclin D1 expression and overall survival (OS).

Ten studies reported data on cyclin D1 expression and OS in bladder cancer. Combined data from all the 10 studies showed that increased cyclin D1 levels were not correlated with OS with a pooled HR estimate of 0.93 (95% CI: 0.83–1.04) (Figure 2). There was a significant degree of heterogeneity (I^2^ = 60.8%, *P* = 0.006). Subgroup analysis indicated that elevated cyclin D1 levels were significantly associated with OS in muscle-invasive bladder patients (HR: 0.95, 95% CI: 0.91–0.99), without significant heterogeneity in the data (I^2^ = 0.0%, *P* = 0.456) (Table 2).

**Table 2:** Subgroup results of OS and heterogeneity test

			**Heterogeneity test**		
**Variables**	**Study number**	**HR (95% CI)**	**Q**	***P***	**I**^**2 **^**(%)**
Total OS	10	0.93 (0.83–1.04)	22.95	0.006	60.8
Region					
Asian	3	1.26 (0.44–3.62)	9.21	0.010	78.3
Caucasian	7	0.93 (0.86–1.02)	13.01	0.043	53.9
Sample size					
>100	5	0.90 (0.55–1.47)	15.35	0.004	73.9
<100	5	0.86 (0.58–1.26)	7.44	0.114	46.2
Follow-up time (month)					
>40	5	0.78 (0.50–1.21)	9.38	0.052	57.4
<40	5	0.98 (0.62–1.55)	13.33	0.010	70.0
Stage					
Superficial	3	0.79 (0.32–1.96)	5.81	0.055	65.6
Muscle-invasive	2	0.95 (0.91–0.99)	0.56	0.456	0.0
All	5	0.92 (0.54–1.55)	16.17	0.003	75.3
Cut-off					
>10%	4	0.87 (0.55–1.39)	6.07	0.108	50.5
≤10%	5	0.98 (0.62–1.55)	13.33	0.010	70.0

### Cyclin D1 expression and PFS in bladder cancer

**Figure 3:**
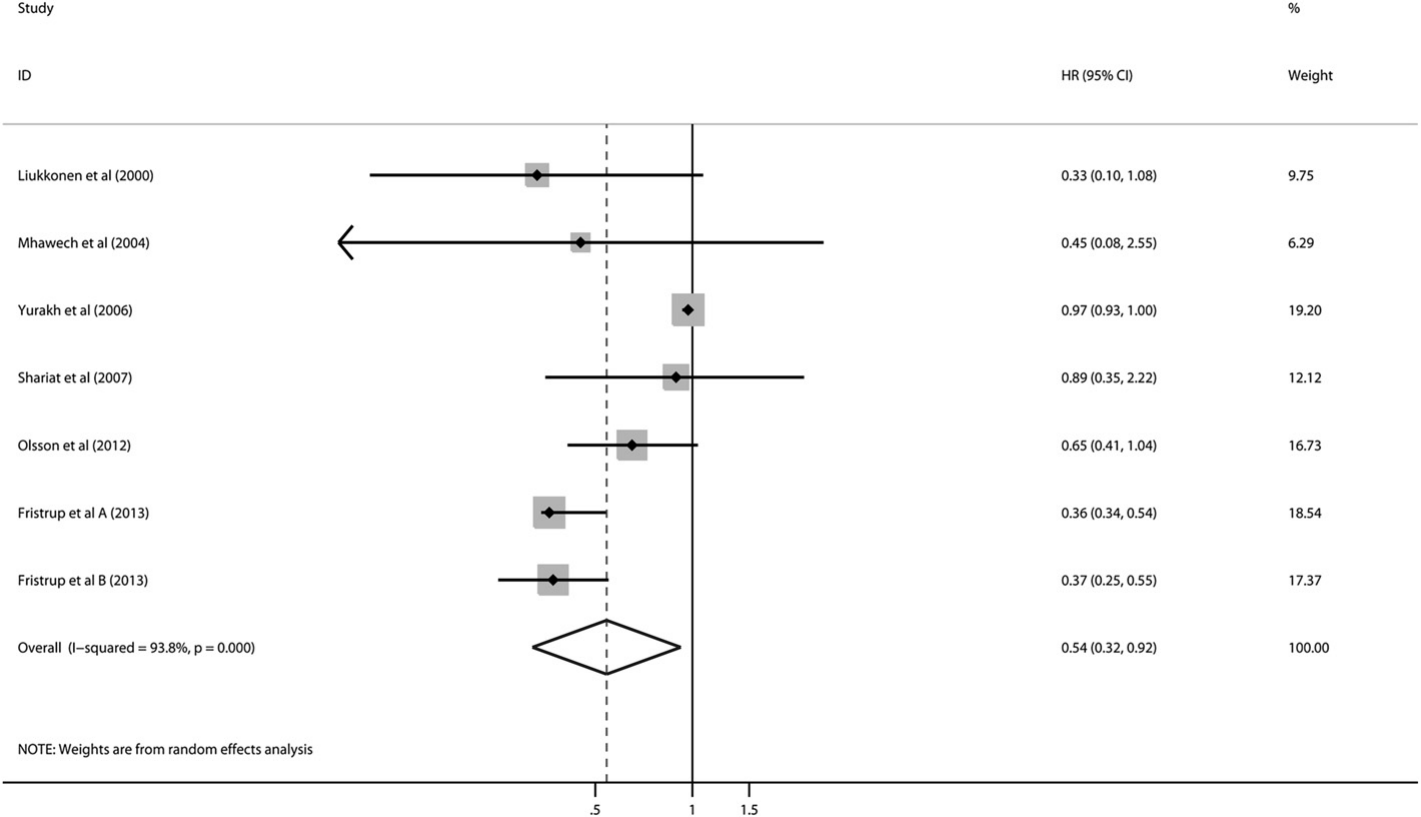
Hazard ratios (HRs) and 95% confidence intervals (CIs) in studies assessing the relationship between cyclin D1 expression and progression-free survival (PFS).

Seven studies reported data on cyclin D1 expression and PFS in bladder cancer. Combined data from all the 7 studies showed that increased cyclin D1 levels were significantly correlated with PFS with a pooled HR estimate of 0.54 (95% CI: 0.32–0.92) (Figure 3). There was a significant degree of heterogeneity (I^2^ = 93.8%, *P* <0.001).

### Cyclin D1 expression and RFS in bladder cancer

Four studies reported data on cyclin D1 expression and RFS in bladder cancer. Combined data from all the 4 studies showed that increased cyclin D1 levels were not correlated with RFS with a pooled HR estimate of 0.94 (95% CI: 0.74–1.19). There was a significant degree of heterogeneity (I^2^ = 71.2%, *P* = 0.015).

### Cyclin D1 expression and DFS in bladder cancer

Two studies reported data on cyclin D1 expression and DFS in bladder cancer. Combined data from these 2 studies showed that increased cyclin D1 levels were not correlated with DFS with a pooled HR estimate of 1.28 (95% CI: 0.22–7.59). There was a significant degree of heterogeneity (I^2^ = 92.0%, *P* <0.001).

### Publication bias

A Begg’s funnel plot was presented for the visual assessment of overt publication bias for the included cohorts in cyclin D1 expression. The funnel plot shapes showed no obvious evidence of asymmetry for OS and PFS. An Egger’s test was then adopted for the formal evaluation (statistical significance was set at *P* <0.05). The *P* value indicated that there was no significant publication bias in OS (*P* = 0.607) and PFS (*P* = 0.093) among these included studies.

## Discussion

This meta-analysis aimed to examine the association between increased cyclin D1 expression and the prognosis of bladder cancer patients. Our analysis combined the outcomes of 2,591 bladder cancer patients from 15 individual studies, indicating that altered cyclin D1 expression was not correlated with OS, RFS, and DFS of bladder cancer patients, but was with PFS (HR: 0.54, 95% CI: 0.32–0.92). Subgroup analysis revealed that increased cyclin D1 expression was also significantly associated with good OS in muscle-invasive bladder cancer patients (HR: 0.95, 95% CI: 0.91–0.99).

Cyclin D1 has been extensively investigated in cancer development and is seen as an important regulator of the G1- to S-phase transition in the cell cycle [28]. In addition, it also has been shown that cyclin D1 mediates DNA repair [29]. Furthermore, cyclin D1 may be an important prognostic indicator for human cancer. Xu et al. found that cyclin D1 overexpression impacts the prognosis of ER-positive breast cancer patients, but not patients with unselected primary breast cancer or patients treated with neoadjuvant chemotherapy [30]. Zhao et al. reported that cyclin D1 expression level detected by IHC is associated with worst clinicopathological features and prognosis for esophageal squamous cell carcinoma [31]. Rainsbury’s study indicated that nuclear cyclin D1 may be a prognostic biomarker of survival in oropharyngeal squamous cell carcinoma [32]. However, in the field of bladder cancer, the search for a prognostic value of cyclin D1 expression has produced different results. Thus, a meta-analysis is essential to achieve a clearer picture of the prognostic value of cyclin D1. To our knowledge, to date, no meta-analysis regarding this relationship has been published.

In recent years, molecular biomarkers have been examined for prognostic assessment despite results remaining inconsistent and controversial. There are no molecular markers that are routinely used in bladder cancer. In the present meta-analysis, our results suggest that overexpression of cyclin D1 is a prognostic factor for good PFS in bladder cancer patients. The clinical implication of this finding is to help us to identify the subjects at high risk of progression after surgery. Patients with lower expression of cyclin D1 may be treated more carefully and followed closely.

Our study had some important strengths. Previous studies have been reported inconsistent and conflicting results about the association between cyclin D1 overexpression and the prognosis of bladder cancer. As individual studies may have insufficient statistical power, our study of 15 studies involving a large number of cases and participants has enhanced statistical power to derive a more precise and reliable estimation of the relationship between them. Nonetheless, several limitations of this meta-analysis should be discussed.

First, although publication bias was not present for OS and PFS, some inevitable publication bias may exist, because only studies published in English and Chinese were included in our meta-analysis. Second, the number of selected studies was still relatively small, and the significant between-study heterogeneity was detected in most comparisons, which may distort the meta-analysis. Third, since some HRs were not directly reported in the studies, we had to calculate them from the data provided in the papers or extrapolate them from the survival curves. The estimated HR might be less reliable than the data obtained directly from published statistics.

## Conclusions

In summary, despite the limitations, results of our meta-analysis suggest that increased cyclin D1 expression is significantly associated with good PFS in bladder cancer. Whether it could be used as a predicative factor for clinical assessment requires large-scale population studies among different ethnicities and regions.

## Abbreviations

CI: Confidence interval; DFS: Disease-free survival; HRs: Hazard ratios; IHC: Immunohistochemistry; OR: Odds ratio; OS: Overall survival; PFS: Progression-free survival; RFS: Recurrence-free survival.

## Competing interests

The authors declare that they have no competing interests.

## Authors’ contributions

SDW and BMR conceived the study concept and participated in its design, data extraction, statistical analysis, manuscript drafting, and editing. WJL participated in the literature research, manuscript drafting, and editing. YPY participated in design and data extraction. All authors read and approved the final manuscript.
